# Progressive Uveitis as a Diagnostic and Therapeutic Challenge in Whipple’s Disease

**DOI:** 10.7759/cureus.72704

**Published:** 2024-10-30

**Authors:** Claire van der Pluijm, Lucie Pothen, Gregoire Wieers, Joachim Van Calster, Alexandra Kozyreff

**Affiliations:** 1 Internal Medicine, Cliniques Saint Pierre, H.uni, Ottignies, BEL; 2 Internal Medicine and Infectious Diseases, Cliniques Universitaires Saint Luc, Brussels, BEL; 3 Institute of Experimental and Clinical Research (IREC), UC Louvain, Brussels, BEL; 4 Medicine, University of Namur, Namur, BEL; 5 Unit of Research in Clinical Pharmacology and Toxicology (URPC), University of Namur, Namur, BEL; 6 Namur Research Institute for Life Sciences (NARILIS), University of Namur, Namur, BEL; 7 Ophthalmology, UZ Leuven, Leuven, BEL; 8 Ophthalmology, Cliniques Universitaires Saint Luc, Brussels, BEL

**Keywords:** hypopyon, immune reconstitution inflammatory syndrome, intermediate uveitis, retinal vasculitis, whipple’s disease

## Abstract

A caucasian male in his 60s presented with a several-month history of weight loss and recurrent fever, accompanied by bilateral sensorineural hearing loss and progressive uveitis. Initial investigations were inconclusive, including Pet CT and duodenal biopsy with *Tropheryma whipplei* polymerase chain reaction (PCR). Based on a suspicion of autoimmune disease, immunosuppressive treatment was initiated without clinical improvement. Whipple disease (WD) was finally disclosed through positive PCR identification of *Trophyrema whipplei* in vitreous biopsy and repeated stool sampling. The patient was treated with intravenous ceftriaxone and doxycycline. After a few days of antibiotics, the patient's visual acuity decreased dramatically with left eye pain and hypopyon, suggesting an immune reconstitution inflammatory syndrome (IRIS) and requiring high-dose steroids. This case report highlights the diagnostic and treatment challenges of Whipple's disease, a rare systemic infection often misdiagnosed due to its nonspecific symptoms.

## Introduction

Whipple's disease (WD) is a rare and potentially fatal systemic infection caused by the gram-positive bacteria *Tropheryma whipplei*. Symptoms are associated with the dissemination of the pathogen from the digestive tract to other tissues, where it can exert its pathogenicity and trigger inflammatory responses [1]. Classic WD diagnosis is challenging due to its heterogeneous nonspecific presentation and is often delayed by years. Misdiagnosis, frequently as inflammatory conditions, often occurs, even after extensive biological investigations and use of the gold standard duodenal biopsy. Subsequent mistreatment with immunosuppressive drugs can expose patients to both severe iatrogenic consequences and disease progression. Following appropriate treatment, immune reconstitution inflammatory syndrome (IRIS) is a severe complication of WD treatment, favored by previous immunosuppressive treatment [1].

## Case presentation

A caucasian male in his 60s was referred for uveitis discovered in the context of persistent inflammatory syndrome, bilateral high-frequency sensorineural hearing loss, and alternating bowel patterns. There were no other symptoms. He reported no history of trauma or exposure to ototoxic drugs. He had a medical history of type 2 diabetes, for which he took metformin. A thorough physical examination was unremarkable.

Two years early, the patient had reported symptoms, including low-grade fever, fatigue, hearing loss, arthralgia in the interphalangeal joints, back pain, and a progressive unintentional weight loss of 8% body weight within a year. At this time, the work-up included fluorine-18 fluorodeoxyglucose positron emission tomography/computed tomography (^18^FDG-PET/CT) and a gastroscopy, which revealed erosive gastropathy. A duodenal biopsy specimen analysis showed no periodic acid schiff (PAS) positive cells, and polymerase chain reaction (PCR) testing for *T. whipplei *in the duodenum was negative. Given the absence of a clear diagnosis, the patient was kept under active surveillance. The fever disappeared, but mild inflammatory syndrome persisted. A few months later, a routine ophthalmological evaluation in the context of type 2 diabetes revealed a new onset of bilateral vitritis with signs of retinal vasculitis.

Facing this progression associated with sustained inflammatory syndrome, another set of tests comprising trans-thoracic echocardiography, 18FDG-PET/CT, cerebral MRI, blood smear, and bone marrow biopsy was performed again and returned normal. Blood tests only revealed anemia and inflammatory syndrome.

Infectious serologies (*Borrelia, Bartonella, Brucella, Babesia, Ehrlichia, *and* Treponema pallidum)* and auto-immune serologies were negative. Urine analysis was normal, repeated blood cultures were sterile, and the interferon-gamma release assay for tuberculous antigens was negative. As intra-ocular inflammation worsened with a decrease in visual acuity, owing to the suspicion of an inflammatory condition (atypical Cogan syndrome or idiopathic bilateral uveitis), oral azathioprine (2mg/kg/d) was combined with an intra-ocular implant of dexamethasone (Ozurdex®), notably to limit the secondary effects of systemic steroids in a type 2 diabetic patient.

**Figure 1:**
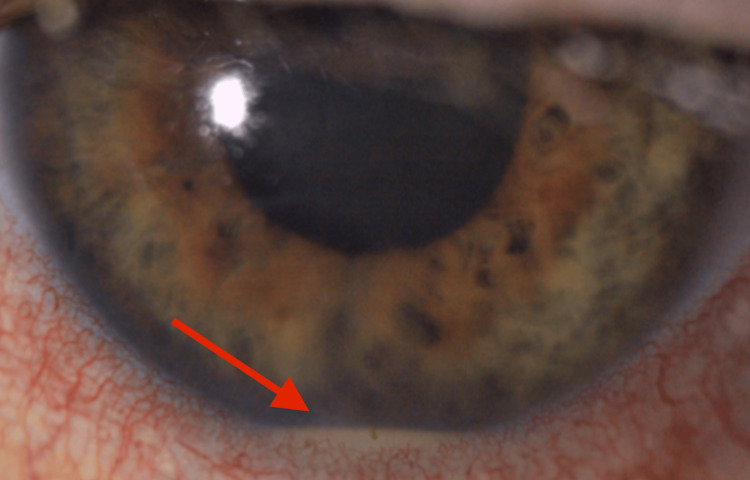
Hypopyon Hypopyon secondary to IRIS, see red arrow

Two months later, ophthalmological follow-up showed worsening of bilateral uveitis with increased vitritis and new atypical cells. A vitreous biopsy was decided to disclose masquerade syndrome and retest for *T.whipplei* PCR. A stool PCR was also performed. Both finally came back positive, confirming the diagnosis of Whipple’s disease (WD). Azathioprine was therefore stopped. Given the central nervous system involvement, intravenous ceftriaxone 2g daily and oral doxycycline 100mg twice daily were started. Six days after antibiotic initiation, vision loss recurred, and new floaters appeared in the left eye, which was also painful. A hypopyon was visible (Figure 1). 

To note, the patient had no fever or inflammatory syndrome anymore. The recent tapering of immunosuppressive drugs combined with antibiotic treatment initiation raised suspicion for immune reconstitution inflammatory syndrome (IRIS). 500mg of intravenous methylprednisolone once daily was administered for three days, followed by one subconjunctival injection of triamcinolone. Antibiotic treatment was maintained twice daily with doxycycline (100mg) and hydroxychloroquine (200mg). Six months later, the patient returned to gardening daily and stabilized his weight, and inflammatory syndrome didn’t reappear. Despite the stagnation of visual acuity at 4/10 of the left eye, mainly due to infection sequelae, vision improved with visual acuity of the right eye rising from 1/10 before antibiotics to 9/10 at the last follow-up visit. 

## Discussion

WD is a rare systemic infection caused by the gram-positive bacteria *Tropheryma whipplei *[1]. Most authors distinguish infection confined to specific tissues as localized WD from disseminated disease, also known as classic WD. In classic WD, patients usually present with the following triad: weight loss (>90%), abdominal complaints (diarrhea, abdominal pain, or malabsorptive syndromes), and long-standing non-destructive arthropathy (70-90%) resistant to conventional treatments [2-4]. Prolonged fever is often present (≤ 50%) [2,3]. In the advanced stages of the disease, all organ systems may be affected, with culture-negative endocarditis, pulmonary infiltrates, or mediastinal lymphadenopathy [1,2]. Granulomatous inflammation has been reported, rendering the differential diagnosis with other granulomatosis challenging [5-7]. In less than half of patients with classical WD, neurological symptoms evolve and represent the most serious manifestations [8]. Although most neurological symptoms are irreversible, hearing loss has been described as partially reversible following antibiotic therapy [9,10].

Whipple's disease diagnosis 

**Figure 2:**
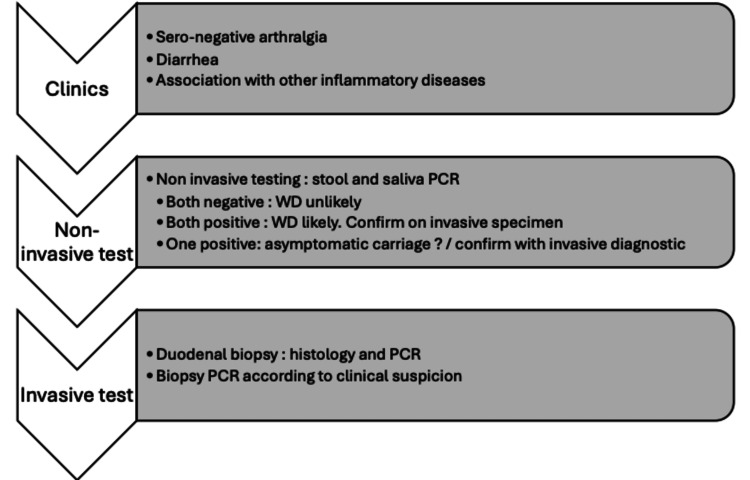
Suggested diagnostic algorithm for Whipple’disease diagnosis adapted from [2]

Due to the rarity of WD, more prevalent alternative diagnoses must be excluded first. Diagnosis relies on histopathological findings compatible with WD and PCR identification of *T. whipplei*. PCR assays target the 16S rRNA or repetitive sequences of *T. whipplei* genes. The latter has shown increased sensitivity and specificity for diagnosis [2,11]. Various authors agree that PCR should be performed on several samples, including invasive ones, whenever possible. Some authors suggest non-invasive screening of *T. whipplei* DNA in feces and saliva or blood samples as the first diagnostic step [2,11] (Figure 2). 

When two non-invasive specimens are positive for *T. whipplei*, diagnosis of WD should be highly considered [2,11]. The second recommended diagnostic step is a duodenal biopsy with periodic acid-Schiff (PAS) staining and PCR testing and, optionally, sampling from other invasive tissues according to the clinical context for PCR and histology [2,11]. Affected tissue will show foamy macrophages containing PAS-positive granules on histology. Negative results (PCR screening and duodenal biopsy) seemingly exclude the classic subset of the disease, but localized WD can still be considered if clinical suspicion is high (Figure 2) [2]. The final diagnosis is retained when at least duodenal PCR is positive with compatible duodenal histological findings or when both biopsy and PCR yield positive results from other tissue samples such as synovial fluid, cerebrospinal fluid, vitreous body, or another symptomatic organ.

Our case first presented with both negative duodenal biopsy and PCR, but WD was later confirmed by* T. whipplei *identification in vitreous biopsy and feces. Delayed diagnosis due to initial WD exclusion based on negative duodenal biopsies are reported. Biesen et al. report a male in his 50s who presented with symmetrical polyarthralgia that predominantly affected the large joints. Rheumatoid arthritis was diagnosed, but prednisolone and methotrexate were therapeutically ineffective. Refractory arthritis was re-evaluated, and WD was excluded based on negative gastro-intestinal histology and PCR. Finally, *T. whipplei* was identified on synovial joint sampling [10]. Crews et al. also reported a case of classical WD with negative duodenal histology and PCR. The diagnosis was based on positive blood PCR and positive PCR on invasive tissue sampling [12]. In Gunther et al.’s case series, 9 patients (5% of their cohort) with classical WD were diagnosed on the grounds of positive extra-intestinal PCR identification of *T. whipplei* after negative duodenal testing [13]. Altogether, these reported cases demonstrate that a negative biopsy and PCR in the duodenum is insufficient to exclude WD.

False-negative duodenal biopsies in WD can be attributed to minimal WD-related lesions, unequal distribution of the bacteria in the intestine, or patients previously treated with anti-TNF inhibitors [14]. In patients without gastrointestinal symptoms, biopsies and PCR testing can also be negative [3]. Finally, false-negative PCR results can be explained by the presence of inhibitors or low amounts of bacteria in the specimen [11].

WD uveitis and immune reconstitution inflammatory syndrome 

IRIS is a dysregulated immune response, resulting in paradoxical clinical and biological deterioration after effective antimicrobial treatment due to the reversal of prior immune suppression [15-18]. IRIS prevalence in WD is as high as 10 to 20% [16,17]. Several presentations have been reported, such as fever, arthralgia, erythema nodosum, small bowel perforation, or uveitis [15-19]. Notably, a mortality rate of 10% has been reported [17], with previous immunosuppressive therapy significantly correlating with IRIS occurrence [17]. The current management of IRIS is based on the empirical use of steroids. Some clinicians also recommend thalidomide [15].

## Conclusions

We presented a case of long-lasting WD with an initial negative workup, complicated by intermediate uveitis, which was finally disclosed by PCR on vitreous biopsy. This case highlights the diagnosis challenge of WD, a rare infectious condition that should be evaluated in patients presenting rheumatological features that do not respond to classical treatment or are associated with digestive symptoms. The diagnosis relies on histopathological findings compatible with WD and PCR identification of *T.whipplei* and sometimes requires PCR testing on multiple non-invasive and invasive samples. In a patient previously mistreated with immunosuppressive therapy, the clinician should also be aware of the significant risk of IRIS at the beginning of the antibiotic treatment.
